# Nanofertilizer Possibilities for Healthy Soil, Water, and Food in Future: An Overview

**DOI:** 10.3389/fpls.2022.865048

**Published:** 2022-05-23

**Authors:** Krishan K. Verma, Xiu-Peng Song, Abhishek Joshi, Vishnu D. Rajput, Munna Singh, Anjney Sharma, Rajesh Kumar Singh, Dong-Mei Li, Jaya Arora, Tatiana Minkina, Yang-Rui Li

**Affiliations:** ^1^Sugarcane Research Institute, Guangxi Academy of Agricultural Sciences, Nanning, China; ^2^Key Laboratory of Sugarcane Biotechnology and Genetic Improvement (Guangxi), Ministry of Agriculture and Rural Affairs, Nanning, China; ^3^Guangxi Key Laboratory of Sugarcane Genetic Improvement, Nanning, China; ^4^Department of Botany, Mohanlal Sukhadia University, Udaipur, India; ^5^Academy of Biology and Biotechnology, Southern Federal University, Rostov-on-Don, Russia; ^6^Department of Botany, University of Lucknow, Lucknow, India

**Keywords:** abiotic stress, bioavailability, environment, growth-production, nanoparticles, plant nutrition, soil

## Abstract

Conventional fertilizers and pesticides are not sustainable for multiple reasons, including high delivery and usage inefficiency, considerable energy, and water inputs with adverse impact on the agroecosystem. Achieving and maintaining optimal food security is a global task that initiates agricultural approaches to be revolutionized effectively on time, as adversities in climate change, population growth, and loss of arable land may increase. Recent approaches based on nanotechnology may improve *in vivo* nutrient delivery to ensure the distribution of nutrients precisely, as nanoengineered particles may improve crop growth and productivity. The underlying mechanistic processes are yet to be unlayered because in coming years, the major task may be to develop novel and efficient nutrient uses in agriculture with nutrient use efficiency (NUE) to acquire optimal crop yield with ecological biodiversity, sustainable agricultural production, and agricultural socio-economy. This study highlights the potential of nanofertilizers in agricultural crops for improved plant performance productivity in case subjected to abiotic stress conditions.

## Introduction

The ever-increasing population and limited cultivable agricultural regions have resulted in new farming agro-technologies to sustain agricultural production and protection worldwide (Rodrigues et al., 2017; Adisa et al., 2019; Rajput et al., 2021a; Verma et al., 2022), as present global human population 7.6 billion may reach 8.6, 9.8 billion by 2030 and 2050, respectively, also projected approximately 11.2 billion by the end of 21st century with serious consequences on world food demand (United Nations, 2017). Growth in affluence and low and middle developed nations is anticipated to expedite a dietary shift away from cereals toward meat, fruits, and vegetables (FAO, 2017; Adisa et al., 2019; Fellet et al., 2021) to produce more food under limited resources (King, 2017; El-Saadony et al., 2021; Rajput et al., 2021a) with loss in larger quantity in developing nations during manufacturing and supply chains due to the unavailability of infrastructure, equipment, and technologies (Kah et al., 2019; Wu and Li, 2022).

Fertilizer consumption increased globally in recent decades with soil nutrient loss (Chugh et al., 2021) due to its cumulative addition to enhance crop productivity (Savci, 2012; Sun et al., 2015; Lin et al., 2019; Verma et al., 2022), with loss in soil health and rising environmental issues (Hasler et al., 2015; Li et al., 2018). Nanoparticles (NPs) may natural or bioengineered with 1–100 nm diameter significantly differ in physical and chemical properties (Rajput et al., 2021b; Verma et al., 2022), available as commercial nanofertilizers (NFs) around the globe, namely, nitrogen (IFFCO Nano Urea, IFFCO, India), phosphorus (TAG Nano Phos, SK Organic Farms, India), potassium (NanoMax Potash, JU Agri Sciences, India), zinc (Geolife Nano Zn, GeolifeAgritech India Ltd., India, SilvertechKimya Sanayi veTicaret Ltd., Turkey, and AFME Trading Group, UK), calcium (Nano Calcium Chelate Fertilizer, AFME Trading Group, UK, Nubiotek®Ultra Ca, Bioteksa, Mexico, Fertile Calcium 25, HPL Agronegocios, Brazil and Lithical, Litho Plant, Brazil), iron, magnesium (Nubiotek®HyperFe+Mg, Bioteksa, Mexico), magnesium, molybdenum, zinc (Nanovec TSS 80, Laboratories, Bio-Medicin, Brazil), silicon (Nano Land Baltic, Lithuania), potassium and phosphorus (Fosvit K30, Kimitec Group, Spain), boran (Nano Bor20%, Alert Biotech, India), and silver (Nano-Ag Answer®, Urth Agriculture, USA) (Dimkpa and Bindraban, 2017; Rajput et al., 2021; Kalwani et al., 2022). NPs facilitate beneficial functions for the nitrogen cycle, enhancing enzyme activities and stimulating soil plant-friendly microbes. Silver NPs have also been shown to increase the density of diazotrophic bacteria in soil, while CuO NPs triggered plant growth-promoting bacteria (PGPR) in the rhizosphere of red sage (*Salvia miltiorrhiza* L.) (Shah et al., 2014; Wei et al., 2021) with beneficial usage of NFs in crop production.

Recent advancements in sustainable agriculture have seen the beneficial usage of various NFs for increased crop production. However, the intentional use of this technology in agricultural activities could have several unforeseen and irreversible consequences (Kah, 2015; Mahapatra et al., 2022). New environmental and unexpected health safety concerns could limit the application of this technology in agricultural crop productivity (Dimkpa and Bindraban, 2017; Ashkavand et al., 2018; Mittal et al., 2020), also in food security (Lopez-Moreno et al., 2018; White and Gardea-Torresdey, 2018; Iqbal, 2019; Rajput et al., 2021). This review provides a better understanding of NFs to encourage interaction among the scientists to expand its application for crop improvement in agriculture. Our review may extend an updated understanding of NFs in crop production/plant productivity.

## Role of NFs in Soil

The application of NFs through soil irrigation ensures double advantages, i.e., soil improvement to optimize plant development productivity (Mahapatra et al., 2022) because the application of larger amounts of inorganic fertilizers to farming land may not be available to plants (Raliya et al., 2018; Tarafder et al., 2020). Therefore, NFs could be a better approach for nutrient absorption by the roots. Various edaphic parameters regulate the range of mineral elements in the soil and may also change microbial colonies and rhizospheric microbial biomass to enhance soil fertility (Huiyuan et al., 2018; Wang et al., 2021), water availability, and plant growth (Mandal and Lalrinchhani, 2021; Rajput et al., 2021a; Verma et al., 2022).

**Figure 1:**
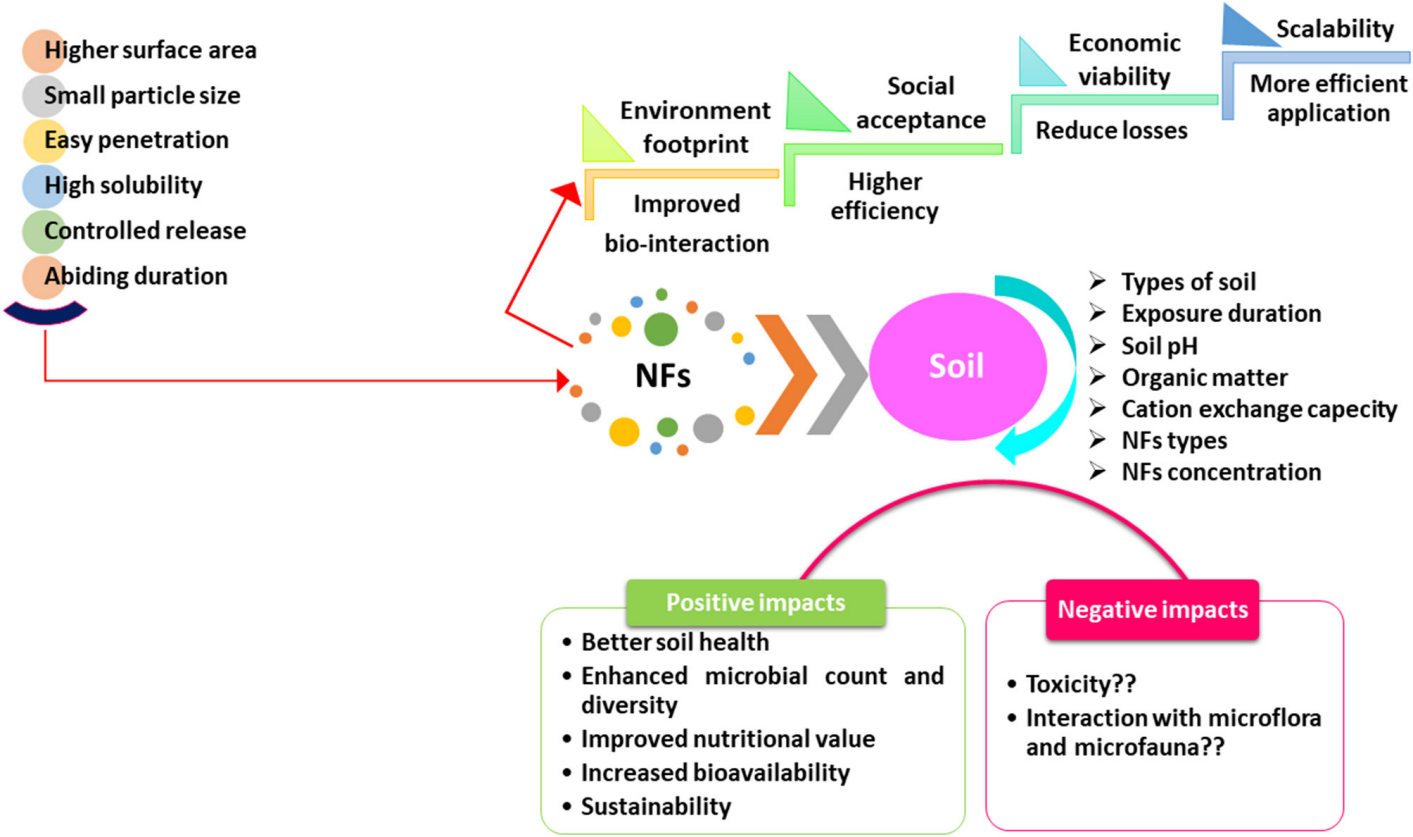
An overview of the role of nanofertilizers in soil.

Roots are a vital interaction site between plants and soil, allowing nutrients, water, and other physiologically important substances to be absorbed (Figure 1), and root development gets influenced by soil aeration, nutrient availability, pH, and soil texture (Taiz and Zeiger, 2010; Adisa et al., 2019; Fellet et al., 2021). The principal mechanism for nutrient accumulation and distribution from the soil to the aerial parts of plant tissues are diffusion and bulk (mass) flow. Diffusion is the transfer of minerals along a concentration gradient from cell to cell (Marschner, 2011), while the bulk flow is found to be the pressure-driven distribution of solutes-water *via* xylem regulated by transpiration and soil nutrient availability (Lawlor et al., 2004; Zulfiqar et al., 2019; El-Saadony et al., 2021). The accumulation of NPs is associated with several ways of absorption/uptake *via* aerial surface, roots, grains, interacting atmospheric variables, rigidity of cell wall, and physiological, anatomical, and biochemical activities of the plant species/cultivars (Rajput et al., 2018a, 2021c; Mittal et al., 2020). The surface tension of NPs on the surface of fertilizer particles is higher than that of ordinary fertilizer, which effectively regulates the release of nutrients (Brady and Weil, 1999; Adisa et al., 2019).

Nanoparticles may get mobilized through apoplastic and symplastic means after entering plants. The apoplastic pathway promotes radial distribution, which moves NPs toward the root's core cylinder and vascular organs and also upward toward aerial portions (Larue et al., 2012; Zhao et al., 2017; Adisa et al., 2019; Verma et al., 2022). The apoplastic pathway is essential for NPs delivery throughout the body, while the Casparian strip inhibits NPs from moving radially in the endodermis of roots, which may be avoided by converting the apoplastic to the symplastic path being a better ordered and regulated way for NPs to travel through the plant body (Palocci et al., 2017; Zhang et al., 2018; Mandal and Lalrinchhani, 2021). Plasmodesmata facilitate cell-to-cell migration once the NPs reach the cytoplasm (Zhai et al., 2014). The smallest particles of TiO_2_ accumulate in plant roots and distribute *via* whole plant tissues without dissolution or crystal phase changes. The NPs of diameter 140 nm or above are no longer accumulated in wheat plant roots, and particles of size 36 nm or above are accumulated in the plant root parenchyma but do not reach the stele to get translocated into the aerial plant parts (Larue et al., 2012). The nanosized NPs during the developmental stages may cause an enhancement of root elongation. TiO_2_ NPs in the range of 1–100 ppm were found to be nontoxic to the soil microbial population, whereas CuO, ZnO, and Ag NPs found to be toxic (Asadishad et al., 2018). Metal NPs may enter seeds and get translocated into seedlings to stimulate plant development *via* seed priming (Sanzari et al., 2019; Seleiman et al., 2021) (Figure 1). NFs may release their nutrients at a slow-release rate, either when applied single or combined with synthetic or organic fertilizers. It may take 40–50 days to release nutrients fully, while synthetic fertilizers do the same in 4–10 days (Seleiman et al., 2021).

## Influence of NFs on Plants

The effects of NFs may be regulated by characteristics of soil, environment, delivery mechanism of NPs, and plant species. The foliar method of entry has proven to be the most effective, as nutrients may be easily absorbed through nanosized pores found in leaf plasmodesmata (Iqbal, 2019; Rajput et al., 2021; Kalwani et al., 2022). NPs also pass-through root hairs (symplastic and apoplastic) to the xylem to reach the stem/leaves (Mittal et al., 2020). The use of NFs induces a considerable rise in the physiological and biochemical indices in crop plants (Rajput et al., 2021a) with improved chlorophyll content/SPAD units in sunflower (Pirvulescua et al., 2015), and also in maize, it is found to be correlated with plant productivity (Morteza et al., 2013; Zulfiqar et al., 2019; El-Saadony et al., 2021) associated with upgraded leaf capacity to capture sunlight, RuBisCO activity, photosynthetic CO_2_ assimilation (Gao et al., 2006; Yang and Hong, 2006; Janmohammadi et al., 2016; Fellet et al., 2021), plant performance, nitrogen metabolism, and soluble proteins.

Zinc-based NFs enhanced peroxidase (POD), catalase (CAT), ascorbate peroxidase (APX), and polyphenol oxidase (PPO) enzymatic responses and proline content in maize (*Zea mays* L.) and cotton (*Gossypium* cultivars) (Weisany et al., 2012; Rezaei and Abbasi, 2014). Foliar application on pearl millet (*Pennisetum glaucum* L.) plants resulted in enhancement of leaf green pigments, soluble protein, and yield (Tarafdar et al., 2014). Applied Zn NPs (15 and 25 nm diameter) enhanced plant length (15%), root length (4%), root diameter (24%), leaf protein (39%), dry mass (13%), and antioxidative enzymatic activities, such as phosphatase (77%), alkaline phosphatase (62%), phytase (322%), and dehydrogenase (21%), with improved crop/grain productivity up to 38% (Tarafdar et al., 2014; Vafa et al., 2015) with stress mitigation during insufficient water, salinity, and nutrient deficiency (Rajput et al., 2021c) because NFs deliver enough nutrients to improve antioxidant activity (Benzon et al., 2015; Fellet et al., 2021; Wu and Li, 2022).

Nanoparticles may interact with respiratory chain enzymes, such as NADH dehydrogenase at low levels, causing the synthesis of uncoupled ATP during respiration. Leakage of the proton and the collapse of the proton motive force may occur if ionic NPs bind to transport proteins (Holt and Bard, 2005; Lok et al., 2006; Adisa et al., 2019). NPs may induce DNA damage indirectly by stimulating reactive oxygen species (ROS), which may affect cross-linking, DNA strand breakage, and sugar or base adducts, among other things (Klaine et al., 2008; Raliya et al., 2018; Mandal and Lalrinchhani, 2021). The replication fidelity of the *rpsL* gene was differently compromised by Ag NPs compared without NPs (Yang et al., 2009).

The production of ROS by NPs is a significant fatal mechanism, and various types of NPs produce various kinds of ROS by decreasing O_2_ molecules (Adisa et al., 2019; Rajput et al., 2021c; Verma et al., 2022). Reactive oxygen species are the byproducts of oxidative cellular metabolism, produced by mitochondria, i.e., hydroxyl radical (OH^−^), superoxide anion radical (O^2−^), hydrogen peroxide (H_2_O_2_), and singlet oxygen (_1_O^2^) (Yin et al., 2012; Fu et al., 2014; Wu and Li, 2022). The chemical makeup of designed NPs determines the amount of ROS produced by NPs (Gonzalez et al., 2008) and the damage of DNA (Zhu et al., 2013; Adisa et al., 2019), which is found to be the biological target of ROS. Oxidative DNA damage includes base and sugar lesions, DNA-protein cross-links, breaks of double and single strands, and the creation of primary sites (Zulfiqar et al., 2019; Mittal et al., 2020). Many studies have demonstrated that ROS plays a vital role in regulating plant cell physiological functions by altering various signaling routes in cell types (Kloepfer et al., 2005; Vara and Pula, 2014; Wehmas et al., 2015; Adisa et al., 2019), while others explored that the size of NPs may be the principal origin of phytotoxicity (Xiao et al., 2015). The application of traditional fertilizer into the soil may impose various disadvantages in terms of plant nutritional bioavailability (Fellet et al., 2021; Mandal and Lalrinchhani, 2021), while foliar application of NFs may be more effective for enhancing overall plant performance/productivity (Roemheld and El-Fouly, 1999; Rajput et al., 2021b) and nutrient use efficiency (NUE) (Abou-El-Nour, 2002; Rajput et al., 2018b; Adisa et al., 2019). Nanocoated compounds with a diameter higher than 10 nm may increase stomatal penetration (Eichert and Goldbach, 2008; Perez-de-Luque, 2017) due to the wide surface area of NFs, which ensures excess sorption capacity and controlled release kinetics with intelligent delivery mechanisms (Rameshaiah et al., 2015; Rajput et al., 2021a,b).

## Significant Role of NFs Against the Environmental Stresses

Plants exposed to NFs show a variety of morphological and physiological alterations, such as germination frequency, lengths of the shoot-root, biomass, chlorophyll fluorescence yield (*Fv/Fm*), photosynthetic efficiency, biomolecules, and cellular injuries, i.e., lipid peroxidation, protein, and cell membrane damage. Several variations have been observed in the plant cell ultrastructures, i.e., disruption of the cell wall, cell membrane, chloroplasts, thylakoids, irregular shape/size of plastoglobules and starch granules, destructive variation in peroxisomes, swollen and damaged mitochondrial cristae, irregular nucleus, rough and thin mesophyll cells, and epidermal, cortical, and stellar cells (Rajput et al., 2018b). Many factors influence the functional expression of NFs, including host plant and specific kinds of NP interaction, surface coating, size, range of concentration, and exposure length (Mittal et al., 2020; Rajput et al., 2021a). The associated mechanisms are yet to be revealed adequately as NPs act excellently for plant performance (Fedorenko et al., 2020; Faizan et al., 2021; Verma et al., 2022), as listed in Supplementary Table 1.

## Drought

**Figure 2:**
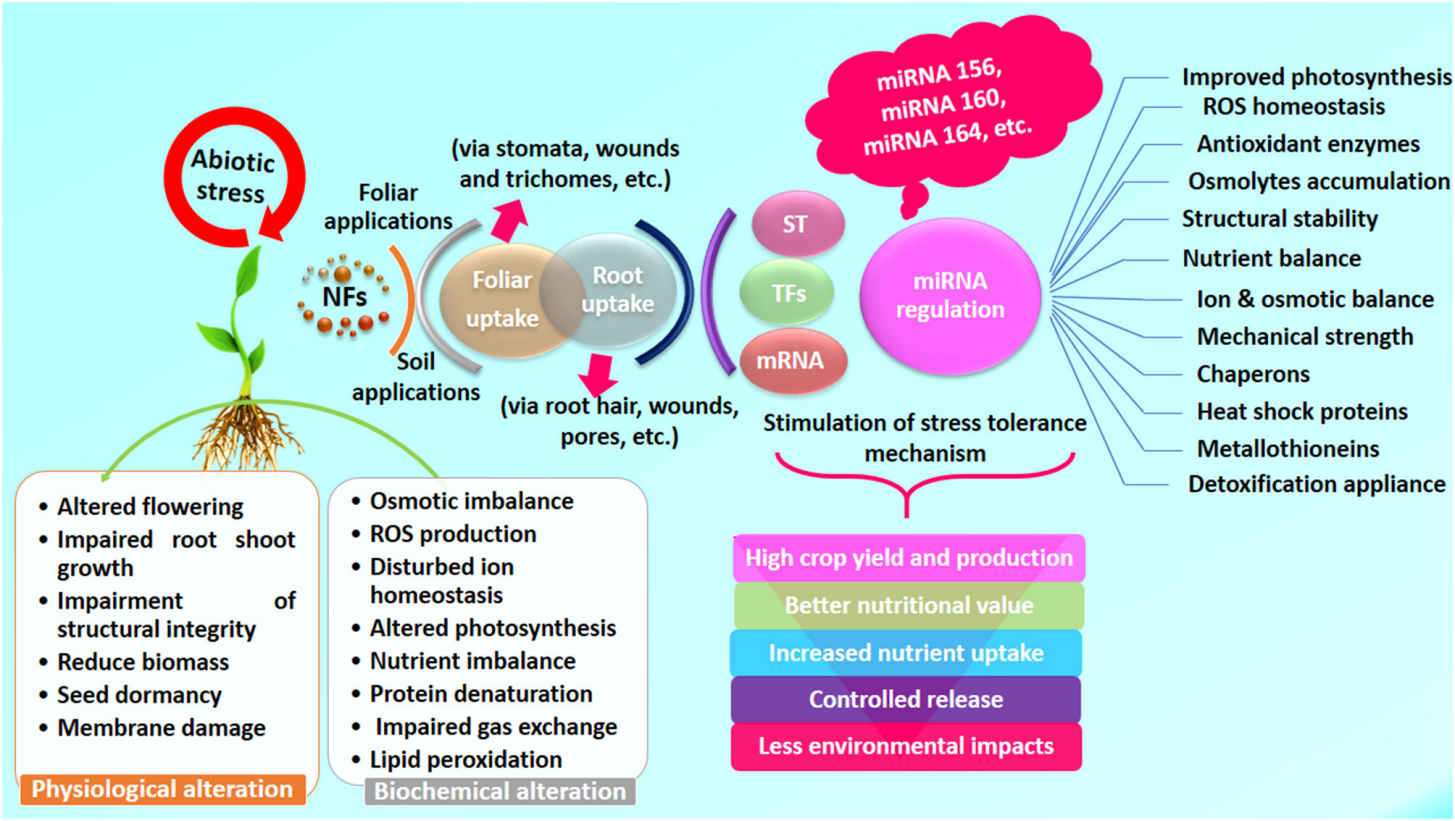
An overview of benefits of NFs to enhance mitigating abilities of plants under environmental stress conditions. ST, signal transduction; TFs, transcription factors.

Water is essential for the transport of nutrients in plants, and its deficiency may cause drought stress that induces morphological, physiological, and biochemical alterations resulting in decreased plant productivity (Kumar et al., 2018b; Desoky et al., 2021). Ghassemi and Farahvash (2018) demonstrated that the foliar use of ZnO NPs (100 ppm) positively affected plant height with increased LRWC and productivity in wheat during anthesis. Mozafari et al. (2018) assessed the feasibility of salicylic acid (SA) and Fe NPs to increase strawberry adaptation strategy to limited water irrigation during the vegetative growth phase with better plant performance and yield. The cotton characteristics and biomass yield during water deficit may be boosted by foliar spray of SiO_2_ (3,200 ppm) and TiO_2_ (50 ppm) (Shallan et al., 2016). Under drought stress, CeO_2_ NPs may increase photosynthetic efficiency (38%), grain yield (31%), and pollen germination (31%) in sorghum. Treated plants of CeO_2_ NPs (10 ppm) decreased superoxide radical (41%), H_2_O_2_ (36%), and MDA (37%) in sorghum leaves subjected to water stress (Djanaguiraman et al., 2018b). Silica NPs may improve the germination efficiency of tomato plants grown during drought conditions (Haghighi et al., 2013; Raliya et al., 2018; Fellet et al., 2021) (Figure 2 and Supplementary Table 1). In contrast, limited water availability resulted in a significant loss in overall plant biomass, including yield (Adisa et al., 2019; Desoky et al., 2021). The use of TiO_2_ NPs boosted wheat plant performance (Jaberzadeh et al., 2013), RuBisCO activity, CO_2_ metabolism, photosynthetic CO_2_ assimilation, and grain productivity (Gao et al., 2006). Under water stress, TiO_2_ NPs enhance the gluten and starch level in wheat, presumably due to the favorable relationship between TiO_2_ and photosynthetic responses (Zhao et al., 2009; Jaberzadeh et al., 2013; Fellet et al., 2021), such as Zn NPs, which favors maize plant productivity (Mittal et al., 2020).

## Salinity

The world's major food crops are threatened by soil salinity (Majeed et al., 2018; Joshi et al., 2020; Rajput et al., 2021a). Plants' ability to absorb water is inhibited under salinity and impairs plant performance (Parihar et al., 2015). Babaei et al. (2017) discovered that seed production increased ca. 17% in treated plants of wheat with Zn-Fe oxide NPs during salinity. By using ZnO-NPs in the callus culture of several tomato cultivars subjected to salinity, the deleterious effects of salt were found to be decreased (Alharby et al., 2016; Adisa et al., 2019). The use of Cu-NPs in tomatoes may improve salt resistance capacity (Hernandez-Hernandez et al., 2018). Farhangi-Abriz and Torabian (2018) and Desoky et al. (2021) applied SiO_2_-NPs to soybeans that improved plant development during salinity with reduced harmful effects of NaCl on bean plants. The root and shoot lengths were significantly increased (23% and 11%) under salinity stress (Alsaeedi et al., 2017) in case cotton was fertilized using Zn-NPs (Hussein and Abou-Baker, 2018). Foliar application of Fe_2_O_3_ and ZnO NPs enhances root growth under salinity in maize and lupine (Latef et al., 2017; Zulfiqar et al., 2019). Silicon NPs increased salinity tolerance capacity in squash plants (Siddique et al., 2014). Under saline conditions, FeSO_4_ NPs may boost sunflower biomass (Torabian et al., 2017), while treatment of TiO_2_ NPs resulted in increased root morphological traits (1.4-fold), stem height (4.8-fold), and biomass (1.2-fold) in maize (Mutlu et al., 2018). Rossi et al. (2016) found that the CeO_2_ NPs applied to rapeseed increased their sensitivity to salinity (Figure 2 and Supplementary Table 1). Abiotic stressors, such as salinity and drought, have harmful effects on plant growth and production worldwide. Crop output is reduced by 50% due to these abiotic stressors (Wang et al., 2003; Rajput et al., 2021b). Plants suffer from a lack of essential minerals, membrane injury, and enzyme inhibition due to ionic and osmotic stressors (Hasanuzzaman et al., 2013a; Adisa et al., 2019). Salinity reduces the plant water availability, nutrient uptake, productivity, and grain/fruit quality (Grattan and Grieve, 1999), while cocultivation of crops with NPs extends better growth and development (El-Saadony et al., 2021) with enzymatic activities of POD, superoxide dismutase (SOD), and CAT which scavenge ROS (Upadhyaya et al., 2015; El-Saadony et al., 2021). Foliar spray of ZnO and Fe_3_O_4_ NPs-containing Hoagland solution may mitigate salinity stress in *Moringa peregrina* (Soliman et al., 2015; Wang et al., 2018; Fellet et al., 2021) with reduced stomatal conductance. The use of SiO_2_ NPs boosted the uptake of N and P while reducing Na buildup in cucumber plants with improved plant performance subjected to salty circumstances (Siddique et al., 2014), as it improves cell wall turgidity, strength, and flexibility (Yassen et al., 2017; Desoky et al., 2021; Rajput et al., 2021a) with enhanced antioxidative enzyme activity and reduced stomatal conductance. The NFs may also be an effective tool for reducing soil toxicity caused by synthetic fertilizers.

## Waterlogging/Flooding

The lack of oxygen in the rhizosphere during flooding stress induces hypoxia, which may experience energy deficiency and increased ethylene (ETH) production-related genes with reduced respiration (Khan et al., 2017; Verma et al., 2021) with impaired vegetative and reproductive development (Komatsu et al., 2012; Verma et al., 2012, 2014; Banti et al., 2013; Khan et al., 2017). The Al_2_O_3_ NPs-responsive proteins were associated with protein synthesis/degradation of glycolysis and lipid metabolism (Mustafa et al., 2015b) to downregulate the operation of the Krebs cycle that strictly occurs under aerobic conditions and impairs major gain of energy which reduces growth and maintenance (Syu et al., 2014). However, Ag NF-applied plants may be less exposed to O_2_ deprivation, which improves overall plant performance (Rezvani et al., 2012). In soybean plants, during the waterlogging situation, a gel-free proteomic approach revealed that Al_2_O_3_ NPs outperformed ZnO and Ag to increase plant development *via* controlling energy metabolism, causing the death of cells (Mustafa et al., 2015a). NPs may play a key role in lowering hypoxic conditions during waterlogging by modifying metabolism and expression of genes, improving plant performance (Supplementary Table 1).

## High Temperature

Heat stress causes changes in plant characteristics, lipid structure, and protein–lipid interactions (Younis et al., 2020). Plants maintain their photosynthetic efficiency and homeostasis as part of their adaptation strategies when subjected to excess light intensities (Nievola et al., 2017; Yue and Yun, 2018; Fellet et al., 2021). Ag and Si NPs boosted root morphological traits (5–5.4%), stem length (22-26%), and other characteristics with protection in wheat plants during temperature stress (Iqbal et al., 2017; Younis et al., 2020). According to the study by Djanaguiraman et al. (2018a), foliar spraying of Se NPs on sorghum grown during high light intensities (38/28°C) increased pollen germination, productivity, and antioxidant enzyme activities as compared with optimum temperature (32/22°C). It decreased oxidant content, protecting plants from the harmful impacts of oxidative damage caused by high-temperature stress. However, heat altered the interactions between plants and NPs, root development in maize plants when ZnO NPs treated, and also enhanced plant performance and APX activity (24%−57%) during excess light (25°C) (Lopez-Moreno et al., 2017). Nano-TiO_2_ significantly reduced the *Fv/Fm* values and relative electron transport rate (ETR) in tomato leaves (Qi et al., 2013). Significant role of TiO_2_ NPs on photosynthesis, stomatal conductance, and transpiration rate in tomato leaves during excess light intensities (Qi et al., 2013; Raliya et al., 2018; Tarafder et al., 2020; Verma et al., 2022) (Supplementary Table 1).

## Freezing Stress

Chilling stress may damage plant cell organelles and tissues (Hasanuzzaman et al., 2013b) due to enhancement in distorted permeability of the cell wall, which induces ion leakage across the membranes and negatively affects germination plant development (Jalil and Ansari, 2019; Mandal and Lalrinchhani, 2021), while plants also adapt freezing resistance capacity (Heidarvand et al., 2011; Jalil and Ansari, 2019). The survival percentage and cold-resistance capacity is the most important factor for describing genotype resistance to low temperature in chickpea plants under field conditions (−10°C for 15 and 30 min) (Heidarvand et al., 2011). The ability of TiO_2_ NPs to reduce the detrimental effects of extremely low temperatures by minimizing the injury caused by ion leakage from the membranes has been demonstrated (Adisa et al., 2019; Rajput et al., 2021a). Chilling stress threatens photosynthesis, a unique and crucial plant carbon assimilation metabolism. It impairs photosystems in a variety of ways, including reduced photosynthetic pigments, transpiration, CO_2_ absorption, and RuBisCO (photosystem enzyme) breakdown (Liu et al., 2012; Mittal et al., 2020). NPs may boost the synthesis of the RuBisCO enzyme (Jalil and Ansari, 2019), the capacity of chloroplasts to absorb light (Ze et al., 2011), and decrease ROS formation in the plant photosystem (Giraldo et al., 2014). The creation of the chlorophyll-binding protein gene expression and RuBisCO, antioxidant enzyme activity, susceptibility to freezing conditions, and chlorophyll content increases in the presence of TiO_2_ NPs in chickpea plants (Mohammadi et al., 2014; Hasanpour et al., 2015; Tarafder et al., 2020). Plants exposed to chilling stress have higher levels of ROS-scavenging enzymes, such as dehydroascorbate reductase (DHAR), glutathione reductase (GR), and monodehydroascorbate reductase (MDAR), as well as increased MeCu/ZnSOD and MeAPX2 genes, resulting in a reduction in oxidative stress, such as the loss of green pigments, MDA, and H_2_O_2_ generation (Xu et al., 2014; Fellet et al., 2021; Seleiman et al., 2021), as shown in Supplementary Table 1.

## Heavy Metal Toxicity

The heavy metal contamination may affect human *via* the food chain (Arif et al., 2019) as Cd, Hg, As, and Pb are among the top 20 toxic heavy metals according to the Agency for Toxic Substances and Disease Registry (ATSDR) and the US Environmental Protection Agency (EPA). Heavy metals endanger food production (Irshad et al., 2020; Javaid, 2020), while NPs may boost seed germination, photosynthetic rate, antioxidant defense system, yield, and plant vigor (Lian et al., 2020; Usman et al., 2020; Wang et al., 2020). Experts agreed using NPs to combat the varied effects of toxic ions on plants (Liu et al., 2018; Rizwan et al., 2018), as CuO NPs (50 and 100 nm) alleviated adverse effects of As on the number of root branches in rice (*Oryza sativa* L. subsp. japonica) plants (Liu et al., 2018) and ZnONPs enhanced wheat plant biomass, nutrients, and reducing Cd toxicity (Rizwan et al., 2018). Under low As concentrations, Fe_3_O_4_ NPs induced a substantial reduction in rice As absorption (Huang et al., 2018). Using Fe NPs, the accumulation of Cr in sunflower roots and shoot growth was reduced (Mohammadi et al., 2018).

Fe_3_O_4_ NPs enhance bioproductivity, photosynthetic electron transport rate (PETR), enzymatic activities, and accumulation of Fe during Ca-deficient soil (Sebastian et al., 2017). Rice (*Oryza sativa* L.) was sprayed with Si NPs reduced Cd (31–65% and 36–61%) content in the upper- and below-ground plant organs. Increased K, Mg, and Fe content in grains and rachises slightly affect Ca, Zn, and Mn (Chen et al., 2018). CeO_2_ NPs inhibited Cd transfer from roots to shoots (70%) in soybean plants (Rossi et al., 2018). The Ce concentration increased in soybean shoot (60%) and reduced in roots (45%). In rice plants, TiO_2_ NPs significantly reduced Cd toxicity and enhanced plant development, photosynthetic efficiency, and reduced Cd uptake and distribution (Ji et al., 2017). Chitosan NPs increase the dry mass (38%), photosynthesis (45%), and chlorophyll index (40%), while a reduction in MDA (24%) and H_2_O_2_ (20%) content were monitored for 4 weeks after seed sowing as compared with control plants in *Solanum lycopersicum* L. (Faizan et al., 2021).

Silicon NPs protect pea plants from the adverse effects of Cr by reducing Cr accumulation and boosting plant performance (Tripathi and Sarkar, 2015; El-Saadony et al., 2021). Foliar application of Si NPs reduced Cd uptake and distribution from soil to roots and increased Mg, Fe, and Zn ions and photosynthetic pigments in rice plants (Wang et al., 2014; Desoky et al., 2020; El-Saadony et al., 2021). Increased MDA and antioxidative enzymatic activities, i.e., SOD, POD, CAT, and reduced GSH concentration, indicated that Cd caused oxidative stress in rice plants (Adisa et al., 2019; Rajput et al., 2021a). In contrast, the treated plants had reduced MDA but enhanced GSH content as well as varied antioxidative enzymatic activities indicating that they were most Cd resistant (Wang et al., 2011). The uptake of Cd was also enhanced from 129 to 508 μg/plant with an increasing concentration of TiO_2_ NPs (100-−300 mg/kg soil). In wheat seedlings, toxic metals, namely, Pb, Zn, Cd, and Cu decreased root development and increased oxidative stress (Mittal et al., 2020). NPs may be helpful to minimize metal phytotoxicity (Zulfiqar et al., 2019; Tarafder et al., 2020; Verma et al., 2022). Singh and Lee (2016) established the role of TiO_2_ NPs in reducing Cd stress and improving soybean plant development, as shown in Supplementary Table 1.

## Nutritional Imbalance

Plants need nutrients for proper development from the soil. During unfavorable environmental conditions, plant nutrient deficit seems to be a limiting factor for plant development. NFs may help to moderate the adverse impacts of synthetic fertilizers (Rajput et al., 2021a) with the added advantage to compensate nutritional deficiencies to allow the plants to develop normally (Bernal et al., 2007; Baloch et al., 2008), as shown in Supplementary Table 1. The nutrient shortage in soils may pose threat to soil profile by lowering nutritional elements for agricultural crops (Khan et al., 2021). Fertilizers are used in large amounts to boost agricultural yield, although more macronutrients are unavailable to plants (Zulfiqar et al., 2019). Consequently, most plants use about half of the applied fertilizers for proper utilization (Mittal et al., 2020; Fellet et al., 2021), generating a long-term negative impact on the agroecosystem. However, overuse of chemical fertilizers may damage soil profile and microflora and disrupt below-ground food webs, resulting in genetic mutations with variations in ecological ecosystems/biodiversity (Solanki et al., 2016; Raliya et al., 2018; Mandal and Lalrinchhani, 2021). Thus, sustainable alternatives may be explored to improve the functional uses of fertilizers in plants with phytoremediation (Pradhan and Mailapalli, 2017; Adisa et al., 2019). The macronutrients–micronutrients regulate plant protection against harmful stresses. Plants' nutritional status may be improved with the application of NPs to boost yields, stress tolerance, and pathogenesis resistance (Zhao et al., 2020; Verma et al., 2022), as shown in Supplementary Table 1.

## UV-Radiation

UV-B radiations are nonionizing and nonphotosynthetically active, enhance ROS production in plant cells, and damage biological functions, including photosynthesis, ultrastructure of chloroplasts, and genomic DNA in plants, having acquired antioxidative defense machinery to counteract harmful UV radiation by accumulating phenolic chemicals (Khan et al., 2017; Rajput et al., 2021a). NPs protect photosynthetic plant systems from UV-B damage by enhancing photosynthetic pigments, upgrading the RuBisCO enzyme, light absorption, photo-transformation, and transmission of light energy, regulating oxidative stress, and absorbing negative UV radiations (Adisa et al., 2019). In contrast, the inclusion of NPs in the plant development media may enhance the detrimental effects of UV light. The application of CuO NPs alone had no adverse effects, but in case combined with UV light may cause an adverse impact on numerous physio-biological features (Regier et al., 2015; Tarafder et al., 2020). The enhancement of POD activity was found significantly in the plants subjected to CuO NPs for 24 h (Regier et al., 2015). Interactive use of Cd telluride-quantum dots (CdTe-QDs) with UV-B radiation reduced enzymatic activities, photosynthetic pigments, and increased DNA injury in wheat plants, followed by programmed cell death as detected by DNA laddering (Chen et al., 2014), as shown in Supplementary Table 1.

## Role of NFs on Crop Productivity and Quality

Nanofertilizers play an important role in physiological and biochemical mechanisms by enhancing the availability of nutrients in crop plants. Nano NPK improves wheat leaf growth by increasing nutrient availability and stomatal dynamics with photosynthetic capacity (Abdel-Aziz et al., 2018; Fellet et al., 2021; Verma et al., 2022), monitored in cotton and pearl millet (Tarafdar et al., 2014). Electron microscopic observations could detect the presence of NPs in the phloem route from leaf–stem–roots (Abdel-Aziz et al., 2018). Zn NF applied to the leaves significantly boosted overall plant performance, including biomass (Vafa et al., 2015), photosynthetic pigments, and enzymatic activities (Rezaei and Abbasi, 2014; Hussein and Abou-Baker, 2018; Seleiman et al., 2021). Zinc may activate enzymes associated with metabolic processes, i.e., glucose and protein metabolism, growth regulators, pollen production, and biological membrane integrity, affecting the synthesis of natural auxin (Alloway, 2008; Rajput et al., 2021d; Wu and Li, 2022). Thus, growth-boosting hormones may get enhanced with the use of nano Zn fertilizer to improve photosynthetic pigments, plant length, biomass, soluble protein, and carbohydrates in maize (Sharifi et al., 2016). TiO_2_ improves plant biomass, nitrogen assimilation, and photo-reduction activities of PS II and electron transport chain (ETC), also scavenging ROS (Morteza et al., 2013; Raliya et al., 2015; Janmohammadi et al., 2016). The aerosol-amended application was found to be more efficient than soil application on the uptake and accumulation of NPs in plants (Raliya et al., 2015) and the growth characteristics, namely, length of plants, branch numbers, grain weight, and biological yield were found to be upregulated (34-38%) using Zn+Fe NFs in pearl millet and sunflower (Drostkar et al., 2016; Sham, 2017) (refer to Figures 1, 2 and Supplementary Table 1).

Nano Zn has an excess surface area-to-volume ratio, which aids in improving Zn absorption and productivity (Khanm et al., 2018). Nano Zn fertilizer requires ten times less than standard ZnSO_4_. Zinc complexed chitosan NPs enhanced Zn content in grains without affecting grain yield and quality, protein content, spikelets per spike, and 1,000 kernel weight (Dapkekar et al., 2018). Pomegranate fruit productivity may increase (21–46%) per plant after foliar use with nano Zn and boron (B). The application of TiO_2_ NPs as a foliar treatment affects the development of barley plants, boosting plant yield, and seed quality (Janmohammadi et al., 2016), while NPs improve fertilizer efficiency and raise grain production (Janmohammadi et al., 2016; Tarafder et al., 2020).

The amendment in TiO_2_ NPs increases plant biomass by upgrading photosynthetic complexes and nitrogen metabolism (Tarafdar et al., 2014; Janmohammadi et al., 2016; Mittal et al., 2020), as photocatalytic activity of TiO_2_ in nanoform extends benefits for maize plant development and seed quality by boosting pigment formation and light energy conversion (Morteza et al., 2013; Raliya et al., 2018). The use of Fe NFs enhanced soybean (*Glycine max* L.) crop production (Sheykhbaglou et al., 2010). Jaberzadeh et al. (2013) demonstrated that nFe boosted seed production relative to normal plants. Manganese (Mn) NPs applied to mung bean improved crop quality (Ghafariyan et al., 2013) with enhanced NUE, pigments, and photosynthetic rate in groundnut nut (Mekkdad, 2017; El-Metwally et al., 2018; Adisa et al., 2019). At the application of 30 ppm, NFs found the highest values of N, P, Fe, Mn, and Zn concentrations in seeds and straw as well as photosynthetic pigments, carotenoids, total carbohydrate, soluble sugars, protein, and seed oil (%) as relative to normal plants (El-Metwally et al., 2018). Plant length, pod numbers, grain weight-number, length of seeds, seed and pod output, and overall biomass of groundnut were found to be enhanced after foliar application of nanochelated molybdenum (Mo) NPs (Fellet et al., 2021).

The use of NFs resulted in higher crop quality than using standard fertilizers in *Arachis hypogaea* (Prasad et al., 2012). Since Zn is associated with photosynthetic processes, synthesis of photosynthetic pigments, and starch creation, carbonic anhydrase boosts the oil content of sunflower seeds (Sham (2017). Zn NFs enhanced the soluble carbohydrates content, increasing the formation of carbohydrates (Sharifi et al., 2016). Groundnut seeds with Zn NFs acquired higher total starch levels, soluble sugars, protein, and oil (Safyan et al., 2012; El-Metwally et al., 2018; El-Saadony et al., 2021), as Zn was found to be associated with the metabolism of carbohydrates, proteins, and phytohormones, particularly indole acetic acid (IAA), which aids in starch synthesis and grain development (El-Metwally et al., 2018; Zulfiqar et al., 2019) (Supplementary Table 1).

## Role of Carbon-Based Nanomaterials on Plant Growth Regulation

Nanoscale, carbon-based nanomaterials (CNMs), including fullerenes, nanodots, NPs, nanotubes, nanohorns, nanobeads, nanodiamonds, and nanofibers, possess novel physiochemical activities, i.e., small surface area, enhanced chemical reactivity, and improved efficiency to enter plant cells with typical surface morphology (Mukherjee et al., 2016; Kumar et al., 2018a; Diez-Pascual, 2021). CNMs have been investigated as drug carrier vehicles and as smart delivery systems in specific areas of nanopharmacology, nanomedicine, public health, etc. (Niazi et al., 2014; Mohajeri et al., 2019) to ensure the availability of delivered drugs appropriately to the specific target site (s) within the cells (Mukherjee et al., 2016; Verma et al., 2019). Therefore, researchers have drawn attention to advanced biological research and bioengineering (Lowry et al., 2019; Chen et al., 2020; Peng et al., 2020; Diez-Pascual, 2021) to acquire plant growth and development optimally linked with plant productivity under adverse environmental variables (Supplementary Table 1).

## Stimulating and Nonstimulating Effects of CNMs on Plants

The bioregulation process of CNMs may produce two contradictory consequences so far. The first may positively affect plant growth and development, while the second may be highly toxic and significantly influence plant biology. At the same time, negatively charged carbon nanotubes with more functional groups may boost seed germination and seedling biomass with activation of water channel proteins based on a series of studies (Khodakovskaya et al., 2011; Villagarcia et al., 2012; Tripathi and Sarkar, 2015; Mukherjee et al., 2016; Diez-Pascual, 2021). The presence and type of carbon nanotubes may influence pesticide availability in lettuce seedlings, as evidenced by the fact that amino-functionalized carbon nanotubes may increase pesticide concentration in roots and shoots, while nonfunctionalized carbon nanotubes may cause the opposite effect (Hamdi et al., 2015; Chen et al., 2020). These days, researchers are advocating concerns with care about using NPs in agriculture to boost crop yields with a quality environment, while increased accretion of NPs in the plant tissue may affect plant growth and physiological responses by inhibiting seed germination, suppressing plant elongation, reducing biomass, and altering expression of genes with the increase in ROS, which induces oxidation of nucleic acids, proteins, and lipids and poses a threat to the biomembrane (Mukherjee et al., 2016; Yang et al., 2017; Shekhawat et al., 2021; Verma et al., 2022). The single-walled carbon nanotubes (SWCNTs) significantly transport and irreversibly localize within the lipid envelope of extracted plant chloroplasts, improving 3-fold higher photosynthetic efficiency compared with an increase in electron transport rate (Giraldo et al., 2014). The harmful effects of CNPs in plants may get mitigated by having a well-developed antioxidant system that includes several nonenzymatic molecules, such as proline, carotenoids, thiols, and enzymatic antioxidants, like APX, CAT, SOD, GPX, GR, and heme-oxygenase, that may scavenge the surplus ROS (Balestrasse et al., 2008; Mahawar et al., 2021). Furthermore, attempts are yet to be put in to explore the mechanism associated with CNM bioregulation.

## Advantages and Disadvantages of NFs

Technological improvements may enhance the production of agro-industrial, physiological, and agronomical essential metallic NPs for making fertilizers with reduced nutritional losses and enhanced NUE with smart delivery systems (Adisa et al., 2019; Fellet et al., 2021). The NPs may be used as NFs on the plants or in the soil to boost fertilizer uptake and utilization to upgrade plant performance (Liu and Lal, 2015; Rajput et al., 2021a,c) with newer possibilities of nanobiotechnology for improved agriculture in years to come by supporting nutrients' delivery system with a targeted approach and multifunctional features (Nair et al., 2010; Adisa et al., 2019) (Figures 1, 2, and Supplementary Table 1). NFs get delivered at slow rates to extend soil health and fertility with nutrient balance by lowering runoff into groundwater and reducing the risk of toxicity (Zulfiqar et al., 2019; Seleiman et al., 2021). Zeolites have a high selectivity for plant minerals and a large specific surface area due to their nanoporous properties, allowing them to be delivered in a slow, controlled, and regular manner as needed by the plants with improved availability (Iavicoli et al., 2017; Rajput et al., 2021a). The treated plants' entire life cycle was found to be reduced ca. 24% shorter than normal fertilized plants (24%) from sowing to maturity (Abdel-Aziz et al., 2016). Innovative fertilizers are currently getting selected as an alternative over the conventional ones (Dimkpa and Bindraban, 2017; Iavicoli et al., 2017) by providing balanced nutrition to combat various environmental variables with significant advantages for physiological fitness and performance of plants/crops as well.

Nanofertilizers may release their nutrients in 6–7 weeks, while synthetic fertilizers do the same within a week. The synthetic urea fertilizer rapidly loses ca. 70% of its N content after field application through leaching and volatilization, leaving <20% available for plants (Kahrl et al., 2010; Seleiman et al., 2021). The chemical fertilizers are indispensable for improving crop productivity, extensively applied through various approaches (Feregrino-Pérez et al., 2018), and while their actual usage may be less than half of the applied amount of fertilizer (Chen and Wei, 2018), the remaining gets leached down to cause water pollution (Liu and Lal, 2015). It has been reported that macronutrient elements, namely, N, P, and K applied to the soil get lost ca. 40–70%, 80–90%, and 50–90%, respectively (Solanki et al., 2016; Chen et al., 2018; Feregrino-Pérez et al., 2018) with water toxicity. Farmers tend to use repeated applications of these fertilizers to achieve desired yields, which may decrease soil health/fertility with the accumulation of salt concentrations in the rhizosphere with impaired plant/crop growth, performance, and productivity (Feregrino-Pérez et al., 2018; Zulfiqar et al., 2019; Verma et al., 2022).

## Future Perspectives

Sustainable global food security seems to be a big issue in times to come. Therefore, innovative/appropriate agricultural practices may be explored to acquire the target of food production under changing climate variables, rising population, and loss of arable land. The precision crop production must be eyed over the application of suitable NPs in diversified agricultural cropping systems using nanoagricultural input to strengthen plants' capabilities to be cultivated in various agroecological zones to address the challenges with opportunities. The comprehensive proteomic and metabolomic approaches are to be unlayered to correlate NPs-induced gene expression profile of crop plants integrated and regulated by the operation of nucleus genome (nDNA), chloroplast genome (cpDNA), and mitochondrial genome (mtDNA), which confers an overall plants' growth, development, physiological fitness/performance, and carbon concentrating metabolism, i.e., photosynthesis linked with phototransformation of light energy using PSII and PSI appears to play a crucial role in regulating photophosphorylation, CO_2_ fixation, and plant productivity, all eventually results to improve agriculture production worldwide through various cropping systems (Zulfiqar et al., 2019; Aqeel et al., 2022; Kalwani et al., 2022; Mahapatra et al., 2022).

The larger NPs can only have direct access to DNA during cell division (Wang et al., 2013; Bhardwaj et al., 2022). Direct genotoxicity (where NPs directly damage the DNA either mechanically or *via* chemical bonding) and indirect genotoxicity (which includes ROS formation, decreased DNA repair, and association with nuclear protein) are the two types of genotoxicity processes for NPs (Karami-Mehrian and De Lima, 2016; Pagano et al., 2022). The most destructive consequence of NPs on plants is DNA damage, which can occur through direct or indirect pathways. The application of NiO NPs revealed direct genotoxicity in the tomato plants where these NPs could directly access the DNA and caused irreversible cell damage. Co_3_O_4_ NPs cause indirect DNA damage in eggplants that lead to apoptosis in plant cells (Faisal et al., 2016). The DNA damage occurs due to degeneration of mitochondrial cristae, peroxisome proliferation, NO generation, and vacuolization. ZnO NPs caused membrane integrity, DNA strand breakage, and chromosomal damage in *Allium cepa* L., *Nicotiana tabacum* L., and *Vicia faba* L. (Faisal et al., 2013; Ghosh et al., 2016; Bhardwaj et al., 2022). In terms of specific effects on plastid (pt) and mitochondrial (mt) DNA, CdS QD exposure induced possible alterations in the organellar genomes at the substoichiometric level, but nanoscale FeOx and ZnS QDs caused a 1- to 3-fold increase in ptDNA and mtDNA copy numbers. NP CeO_2_ did not alter ptDNA and mtDNA stoichiometry. These results suggest that modification in stoichiometry is a potential morpho-functional adaptive response to NPs exposure caused by variations of bioenergetic redox balance, which reduces the photosynthesis or cellular respiration rate (Karami-Mehrian and De Lima, 2016; Pagano et al., 2022).

The plant–NP interactions in the field must be carefully examined at the molecular level to minimize phytotoxic effects to sustain the soil health, which may boost crop productivity and extend the ecofriendly ecosystem by discouraging huge application of conventional fertilizers. Therefore, our insight for nanoformulation and its application must be focused on soil and groundwater based on innovative, safe, and cost-effective updated interventions for agroecological sustainability to feed the future generations to ensure quality human resources. All these intrinsic abilities of plants may be made to understand the possibility of sustainable crop improvement to fulfill the need for healthy food for all future generations globally.

## Author Contributions

KV, X-PS, and Y-RL conceptualized and validated the study. KV and Y-RL contributed to methodology. KV contributed to software and writing the original draft. KV, X-PS, AJ, VR, AS, RS, D-ML, and JA contributed to formal analysis. X-PS and Y-RL contributed to investigation, visualization, supervision, project administration, and funding acquisition. KV and X-PS contributed to resources. KV and AJ contributed to data curation. MS, TM, and Y-RL contributed to writing, reviewing, and editing the manuscript. All authors approved the manuscript for publication.

## Funding

This research was financially supported by the Guangxi Innovation Teams of Modern Agriculture Technology (nycytxgxcxtd-2021-03), the Youth Program of National Natural Science Foundation of China (31901594), the National Natural Science Foundation of China (31760415), the Guangxi Natural Science Foundation (2021GXNSFAA220022), the Fund of Guangxi Academy of Agricultural Sciences (2021YT011), and Guangxi Key Laboratory of Sugarcane Genetic Improvement Project (21-238-16-K-04-02).

## Conflict of Interest

The authors declare that the research was conducted in the absence of any commercial or financial relationships that could be construed as a potential conflict of interest.

## Publisher's Note

All claims expressed in this article are solely those of the authors and do not necessarily represent those of their affiliated organizations, or those of the publisher, the editors and the reviewers. Any product that may be evaluated in this article, or claim that may be made by its manufacturer, is not guaranteed or endorsed by the publisher.
