# Supplementary material for: Nanofertilizer Possibilities for Healthy Soil, Water, and Food in Future: An Overview
Source: Front Plant Sci. 2022 May 23;13:865048. doi: 10.3389/fpls.2022.865048 (PMC9168910; doi:10.3389/fpls.2022.865048)
Supplement: Supplementary file 1 [file Table_1.docx]

**Nanofertilizer possibilities for healthy soil, water and food in future: an overview**

**Supplementary Table 1.** Role of nanofertilizers on sustainable agriculture

| Stress condition | Plant | NFs | Concentration range | Impacts | Source |
| --- | --- | --- | --- | --- | --- |
| Drought | Soybean  (*Glycine max* (L.) Merr.) | nCeO_2_ (+ charge) /PVP-CeO_2_ (-charge) | 100 ppm | Significantly enhanced efficiency of leaf gas exchange. Both NPs enhanced plant growth activities and yield. | Cao et al., 2018 |
| - | Radish  (*Raphanus sativus* L.) | nCeO_2_ | 0 – 100 ppm | Enhanced plant weight, root area expansion (2%), photosynthetic pigments (12 – 13%), and enzyme activities in plant organs, i.e., roots and leaves | Gui et al., 2017 |
| - | Rice  (*Oryza sativa* L.) | nCeO_2_ | 500 ppm | Rice grains decreased the Fe, S, prolamin, glutelin, lauric, valeric acids, starch and K, Ca, Na, protein albumin, and total sugars upregulated | Rico et al., 2013 |
| - | Wheat  (*Triticum aestivum* L.) | nCeO_2_ | 0 - 500 ppm | The 1-generation (G) decreased the uptake of Ce, Fe, and Mn in plant roots. Treated plants in both generations found seeds with minimum Mn, Ca, K, Mg, and P content compared to 2G stressed plants. 1-G affects the plant morpho-physiological responses and nutritional profile of the 2-G plants | Rico et al., 2017 |
| - | Lettuce  (*Lactuca sativa* L.) | nCeO_2_ | 50-1000 ppm | Excess application of NPs significantly decreased plant development, yield, and enzymatic activities disrupted | Gui et al., 2015 |
| - | Maize  (*Zea mays* L.) | nCuO | 0.02–8 ppm | Improved plant growth parameters (51%), increased Cu uptake and accumulation. The activity of glucose-6-phosphate dehydrogenase was effectively enhanced in foliar and soil irrigation. nCu also boosted the growth and associated enzymes in maize plants. | Adhikari et al., 2016 |
| - | Soybean  (*Glycine max* L.) | nCuO | 50-500 ppm | The size and range of NPs treatment depend on the severity of the toxicity. Changed antioxidative biomarkers with NPs application. | Yusefi-Tanha et al., 2020 |
| - | Tomato  (*Solanum*  *lycopersicum* L.) | nCuO | 10–500 ppm | Slightly enhanced the photosynthetic pigments and sugar content at 10 ppm. Then, these parameters declined after increasing the NPs. Enhanced enzymatic activities, | Singh et al., 2017 |
| - | Cauliflower  (*Brassica oleracea* var. botrytis) | nCuO | 10–500 ppm | Growth parameters, leaf chlorophyll index, and soluble sugar level were enhanced (10 ppm) relative to normal plants—application-dependent upregulation found in enzymatic responses. | Singh et al., 2017 |
| 1. Cu toxicity | 1. Barley 2. (*Hordeum sativum* distichum) | nCuO | 1000 ppm | Loss in germination rate, time, growth, development, changes in root morphological behavior, biomass, maximum chlorophyll fluorescence yield of PSII, and photosynthetic efficiency. nCuO affect the root, stomatal and cellular ultrastructure. The Cu content was found higher than normally grown plants. | Rajput et al., 2018a |
| - | Kidney bean  (*Phaseolus vulgaris* L.) | nCu/ kinetin | 50, 100 ppm | Chlorophyll (22-30%) and minerals as Ca, Mn, and P (33-97%) were decreased, and enhanced root Cu uptake | Apodaca et al., 2017 |
| - | Maize  (*Zea mays* L.) | nCu-chitosan | 400-1200 ppm | Increased growth and development by enhancing the activities of α-amylase and starch levels. | Saharan et al., 2016 |
| - | Tomato  (*Solanum*  *lycopersicum* L.) | nCu–chitosan-PVA | 0.02–10 ppm | Enhanced solid content, titratable acidity, lycopene (37%), and enzymatic activities in fruits (10%). Significantly increased plant growth responses and fruit quality. The enhancement was found in leaf area expansion (17%), clusters (13%), and roots dry mass (30%). | Hernandez et al., 2017 |
| Drought | Barley  (*Hordeum vulgare*L.) | nChitosan | 30-90 ppm | Enhanced LRWC, the weight of seeds, seed protein, proline, SOD, and CAT activities | Behboudi et al., 2018 |
| - | Wheat  (*Triticum aestivum* L.) | nChitosan-NPK | 10–100 ppm | Enhanced the plant performance, harvest/crop index, and yield production concerning normal plants. | Abdel-Aziz et al., 2016 |
| As toxicity | Maize  (*Zea mays* L.) | nSiO_2_ | 10 ppm | Reduce metal toxicity and uptake and increase the ascorbate-glutathione cycle. It is more effective to mitigate metal toxicity in maize plants | Tripathi et al., 2016 |
| Salinity | Strawberry  (*Fragaria x ananassa*) | nSiO_2_ | 50-100 ppm | Development of vegetative growth phase enhanced, upregulated photosynthetic pigments, LRWC (%), canopy temperature (54%), and proline content (81%) | Avestan et al., 2019 |
| Salinity | Tomato  (*Solanum lycopersicum* L.) | nSiO_2_ | 30-180 ppm | Stress-related genes were noted as up (AREB, TAS14, NCED3 & CRK1) and downregulated (RBOH1, APX2, MAPK2, ERF5, MAPK3 & DDF2) to assist in the alleviation of stress | Almutairi, 2016 |
| - | Cotton  (*Gossypium* spp.) | nSiO_2_ | 10 -2000 ppm | Reduced plant growth biomass and affected the nutrient elements, i.e., Cu, Mg in shoots, and Na in roots. SOD and IAA content were significantly affected, SiNPs were found in the xylem sap, and roots transported from roots-shoots via xylem sap. | Le et al., 2014 |
| Drought | Barley  (*Hordeum vulgare* L.) | nSiO_2_ | 125 – 250 ppm | Increased plant morpho-physiological, chlorophyll content (17%), enzymatic, and metabolic activities. | Ghorbanpour et al., 2020 |
| Drought | Cotton  (*Gossypium* spp.) | nSiO_2_ | 3200 ppm | Maintained plant performance and productivity. | Shallan et al., 2016 |
| Salinity and drought | Banana  (*Musa acuminate* L.) Grand Nain | nSiO_2_ | 200-600 ppm | Improved plant development, photosynthetic efficiency (23%), balance K^+^/ Na^+^ ratio (14%), and reduced cell damage injury. | Mahmoud et al., 2020 |
| Freezing | Sugarcane (*Saccharum officinarum* L.) | nSio_2_, nZnO, nSe and Graphene nanoribbons (GNRs) | 5-15 nm, < 100 nm, 100 mesh & 2-15 µm x 40-250 nm | NPs decreased the negative effects of freezing by upgrading the chlorophyll fluorescence yield (*Fv/Fm*), maximum photo-oxidizable PSI (Pm), and photosynthesis. The applied NPs enhanced the light-harvesting pigments in treated plants. The highest carotenoid increased the nonphotochemical quenching of photosystem II. | Elsheery et al., 2020 |
| Heat | Wheat  (*Triticum aestivum* L.) | nSiO_2_ | ~ 100 ppm | Potentially restored the cellular ultrastructural distortions of the organelles such as chloroplast and the nucleus—enhanced photosynthesis as revealed by enhancement in PS II and chlorophyll content efficiency. The loss in MDA content significantly correlated with the cell membrane stability index. | Younis et al., 2020 |
| UV-B | Wheat  (*Triticum aestivum* L.) | nSiO_2_ | 10 ppm | The improved antioxidative enzymatic machinery | Tripathi et al., 2017 |
|  | Spinach  (*Spinacia oleracea* L.) | nFe_2_O_3_ | 100–200 ppm | Plant biomass and Fe accumulation upregulated concerning normal plants. | Jeyasubramanian et al., 2016 |
| Salinity | Peppermint (*Mentha piperita* L.) | nFe_2_O_3_ | 10-30 ppm | Decreased MDA and proline content and enhanced antioxidative enzymatic activities. An appropriate dose of NPs could be used for stress tolerance strategies | Askary et al., 2017 |
| - | Soybean  (*Glycine max* L.) | nFe_2_O_3_ | 30-60 ppm | Positively enhanced the plant developmental stages and chlorophylls. | Ghafariyan et al., 2013 |
| - | Peanut  (*Arachis hypogaea* L.) | nFe_2_O_3_ | 0-1000 ppm | Upgraded plant development and dry mass, leaf chlorophyll index plant hormones, enzymatic activities, and Fe uptake. | Rui et al., 2016 |
| - | Soybean  (*Glycine max* L.) | nFe_2_O_3_ | 0-1000 ppm | Enhanced plant biomass, productivity (48%), and Zn accumulation | Sheykhbaglou et al., 2010 |
| - | Corn  (*Zea mays* L.) | γ- Fe_2_O_3_ NPs | 20 ppm | Improved germination (27%), vigor index (40%), frequency, time, and root development (12%). Minimum concentration of NPs showed significant impacts on plant performance | Li et al., 2016 |
| - | Peanut (*Arachis hypogaea* L.) | γ- Fe_2_O_3_ NPs | 2 ppm | Upgraded photosynthetic CO_2_ assimilation rate and plant productivity | Alidoust and Isoda, 2013 |
| Flooding | Soybean  (*Glycine max* L.) | nAl_2_O_3_ | 50 ppm | Enhanced length of seedlings and upregulated mitochondrial membrane protein | Mustafa and Komatsu, 2016 |
| Salinity | Tomato  (*Solanum esculentum* Mill.) | nTiO_2_ | 0 - 40 ppm | Enhanced the carbonic anhydrase, NR, SOD, POX, accumulation of proline, glycine betaine, growth, and biomass activities. | Khan, 2016 |
| - | Barley  (*Hordeum vulgare* L.) | nTiO_2_ | 0 - 1000 ppm | Improved amino acids (37-51%) and crude protein. Zn and Mn accumulation levels increased. | Poscic et al., 2016 |
| Drought | Linum  (*Linum usitatissimum* L.) | nTiO_2_ | 10-500 ppm | Enhanced green pigments and carotenoids levels, reduced H_2_O_2_ and MDA content | Aghdam et al., 2016 |
| Drought | Wheat  (*Triticum aestivum*L.) | nTiO_2_ | 100-300 ppm | Plant performance, productivity, and gluten content improved | Jaberzadeh et al., 2013 |
| Drought | Wheat  (*Triticum aestivum* L.) | nTiO_2_ | 500-2000 ppm | Increased plant biomass, LRWC, activities of antioxidative enzymes, leaf pigments, and photosynthetic efficiency | Faraji and Sepehri, 2020 |
| Freezing | Chickpea  (*Cicer arietinum* L.) | nTiO_2_ | 2-10 ppm | Reduced membrane injury index and MDA contents | Mohammadi et al., 2013 |
| - | Peppermint (*Mentha piperita* L.) | nTiO_2_ | 0-250 ppm | Improved the growth, biomass, productivity (105%), leaf gas exchange (24%), Fv/ Fm (7%), photosynthetic pigments, and enzymatic and non-enzymatic activities, i.e., nitrate reductase (18%) and carbonic anhydrase (19%) | Ahmad et al., 2018 |
| 1. - | 1. Cucumber (*Cucumis sativus* L.) | nFe_3_O_4_ | 50 – 2000 ppm | Increase plant fitness, yield, and enzymatic capacities such as SOD and POD. Applied NPs enhance/ balance the proper nutrient management to overcome food security and safety. | Konate et al., 2018 |
| 1. Drought | 1. Strawberry (*Fragaria x ananassa* Duch.) | nFe_3_O_4_ | 0.8 ppm | Increased plant performance | Mozafari et al., 2018 |
| 1. - | 1. Ryegrass (*Lolium perenne* L.) | nFe_3_O_4_ | 30-500 ppm | Enhanced enzymatic and non-enzymatic activities, such as SOD, CAT, and MDA | Wang et al., 2011 |
| 1. - | 1. Barley 2. (*Hordeum vulgare* L.) | nFe_3_O_4_ | 125-1000 ppm | Increasing the concentration of nFe_3_O_4­_ was significantly enhanced the plant growth parameters with biomass (leaf-19%, root-88%). Even at applied excess quantity, no toxic effects were observed—significantly increased photosynthetic pigments, soluble protein, and no. of chloroplasts. Higher range of NPs reduced CAT and H_2_O_2_ content. Dramatic variations were found in the photosynthetic genes such as *Pet*A, *psa*A, BCA, and *psb*A. | Tombuloglu et al., 2019 |
| - | Common bean  (*Phaseolus vulgaris* L.) | nAg | 0 – 60 ppm | Enhanced growth traits, biomass, productivity, balance plant hormones, and overall plant performance against normal plants | El-Batal et al., 2016 |
| Heat | Wheat  (*Triticum aestivum* L.) | nAg | 25-100 ppm | Upgraded length of stem (22%), roots (5%), leaf area (34%) and growth parameters, i.e. fresh (2%) & dry weight (0.60%) | Iqbal et al., 2019 |
| Salinity | Fenugreek (*Trigonella foenum-graecum* L.) | nAg | 10-40 ppm | Enhanced germination percentage, biomass, and productivity | Hojjat and Kamyab, 2017 |
| Flooding | Saffron  (*Crocus sativus* L.) | nAg | 40 - 120 ppm | Enhanced growth characteristics with biomass yield | Rezvani et al., 2012 |
| Metal | Cucumber (*Cucumis sativus* L.) | nAg | 300 ppm | Enhanced Ag content and growth and yield/ fruit capacity and quality | Shams et al., 2013 |
| - | Lettuce  (*Lactuca sativa* L.) | nAg | 100 ppm | No phytotoxic symptoms were monitored. Ag significantly trapped on plant leaves. Entrapment of Ag-NPs penetration in the leaves by the stomatal cuticle. | Larue et al., 2014 |
| 1. - | 1. Soybean 2. (*Glycine max* (L.) Mell.) | nAg | 31.2 – 62.5 ppm | Downregulated plant performance and fixation of N. | Ma et al., 2020 |
| 1. Heat | 1. Sorghum 2. (*Sorghum bicolor* (L.) Moench) | nSe | 10 ppm | Enhanced antioxidative defense systems and changes ultrastructural cell organelles | Djanaguiraman et al., 2018a |
| 1. Low and high temperature | 1. Tomato (*Lycopersicum esculentum* Mill.) | nSe | 1-12 ppm | Increased plant growth, development, and productivity | Haghighi et al., 2014 |
| Heat | Corn  (*Zea mays* L.) | nZnO | 50–1600 ppm | At various temperatures, nZnO enhanced the root growth (50%) morphology, Zn uptake, and APX activity (57%) | Lopez-Moreno et al., 2017 |
| - | Lettuce  (*Lactuca sativa* L.) | nZnO | 1–100 ppm | Upgraded plant performance and photosynthesis, Zn uptake as relative to normal plants | Xu et al., 2018 |
| Mineral toxicity | Cotton  (*Gossypium hirsutum* L.) | nZnO | 25–200 ppm | Positively enhanced plant growth (130%) and total biomass (131%), photosynthetic pigments (141%), carotenoids (139%), protein (179%), and antioxidant enzymes, i.e., POX (183%), SOD (264%) and reduced MDA content (68%). | Venkatachalam et al., 2017 |
| - | Pea  (*Pisum sativum* L.) | nZnO | 250 - 1000 ppm | Enhance Zn level in roots and grains. Increased photosynthetic pigments, carotenoid, sucrose content, and overall plant development. | Mukherjee et al., 2016 |
| - | Pearl Millet  (*Pennisetum americanum*) | nZn | Particle size 15 – 25 nm | Positively improved growth, biomass, pigments, and biochemical activities | Tarafdar et al., 2014 |
| - | Tomato (*Lycopersicum esculentum* Mill.) | nZnO | 2–16 ppm | Positively enhanced the plant performance, photosynthesis with carbonic anhydrase, and antioxidative enzyme activities of concentration- and time-dependent variables relative to normal plants. | Faizan et al., 2018 |
| - | Thalecress (*Arabidopsis thaliana* L.) | nAu | 10 - 80 ppm | Improve germination efficiency, growth rate, free radical scavenging responses. Overall plant productivity enhanced | Kumar et al., 2013 |
| Salinity | Cucumber (*Cucumis sativus* L.) | Mn_3_O_4_ | 20, 100 ppm | Increase plant performance, photosynthetic level, photosynthetic CO_2_ assimilation rate, and yield biomass. Improve endogenous antioxidative defense systems. | Lu et al., 2020 |
| - | Black-eyed peas (*Vigna unguiculata* L.) | nMg | 0.5 ppm | Enhanced photosynthetic performance and yield parameters | Delfani et al., 2014 |
| - | Mung bean  (*Vigna radiata* L.) | Quantum dots | 50-75 ppm | Enhanced photosynthetic capacity by enhancing electron transfer rate (ETR) in thylakoid membranes | Chandra et al., 2014 |
| Control condition | Arabidopsis (*Arabidopsis thaliana* L.) | Functional carbon nanodots (FCNs) | 200-500 ppm | The positive correlation was found between the physiological traits of plants and the surface chemistries of NMs. The raw FCNs present maximum promotion capacity in plants biomass and length of roots, and the quantum-sized FCNs are easier to be absorbed by plants and generate more significant impacts on plants | Chen et al., 2020 |
| - | Lettuce (*Lactuca sativa* L.) | FCNs | 104-1750 ppm | Decreased length of roots at long-term duration | Canas et al., 2008 |
| - | Bitter melon  (*Momordica charantia* L.) | Fullerol [C_60_(OH)_20_] | 943-47200 ppm | Enhanced plant growth, yield (54%), water content (24%), fruit number (59%), length (20%) and weight of the fruits (70%), increased anticancerous plant medicines, such as cucurbitacin-B (74%) and lycopene (82%), and antidiabetic plant medicines, i.e., charantin (20%) and insulin (91%) | Kole et al., 2013 |
| - | Corn (*Zea mays* L.) | Fullerenes C_60_ | 500 ppm | Plant biomass decreased (37%) | Torre-Roche et al., 2013 |
| - | Cabbage (*Brassica oleracea* L.*)*, Carrot (*Daucus carota* L.), Lettuce (*Lactuca sativa* L.), Onion (*Allium cepa* L.), Tomato (*Solanum lycopersicum* L.) | FCNs (single-walled) | 9, 56, 315, 1750 ppm | No effect | Canas et al., 2008 |
| - | Rice (*Oryza sativa* L.*)* | Single-walled carbon nanotube | 400 ppm | Reduces growth, productivity, and delayed flowering | Lin et al., 2009 |
| - | Mung (*Vigna radiata* L.) | CNPs | 25-200 ppm | Significantly increase growth, biomass (1.2 fold), yield, chlorophyll content (2 fold), protein (1.14 fold), proline, and antioxidative enzyme activities, such as SOD, GPX, APX, enhance stress-resistance capacity and phytoremediation efficiency of plants in the contaminated soil and/ or environment | Shekhawat et al., 2021 |
| - | Wheat (*Triticum aestivum* L.), Maize (*Zea mays* L.), Peanut (*Arachis hypogaea* L.), Garlic (*Allium sativum* L.) | MW-CNTs | 0-50 ppm | The significant effects were found in the root and shoot development of plants. The low concentration of CNTs was more efficient for plant performance | Srivastava and Rao, 2014 |

**References**

Abdel-Aziz, H.M., Hasaneen, M.N., Omer, A.M. (2016). Nano chitosan-NPK fertilizer enhances the growth and productivity of wheat plants grown in sandy soil. *Span. J. Agric. Res*. *14*, 17. doi: 10.5424/sjar/2016141-8205

Adhikari, T., Sarkar, D., Mashayekhi, H., Xing, B. (2016). Growth and enzymatic activity of maize (*Zea mays* L.) plant: solution culture test for copper dioxide nano particles. *J. Plant Nutr. 39*, 99–115. doi: [10.1080/01904167.2015.1044012](https://doi.org/10.1080/01904167.2015.1044012)

Aghdam, M.T.B, Mohammadi, H., Ghorbanpour, M. (2016). Effects of nanoparticulate anatase titanium dioxide on physiological and biochemical performance of *Linum usitatissimum* (Linaceae) under well-watered and drought stress conditions. *Braz. J. Bot. 39*, 139–146. doi: 10.1007/s40415-015-0227-x

Ahmad, B., Shabbir, A., Jaleel, H., Khan, M.M.A., Sadiq, Y. (2018). Efficacy of titanium dioxide nanoparticles in modulating photosynthesis, peltate glandular trichomes and essential oil production and quality in *Mentha piperita* L. *Curr. Plant Biol.* *13*, 6–15. doi: [10.1016/j.cpb.2018.04.002](https://doi.org/10.1016/j.cpb.2018.04.002)

Alidoust, D., Isoda, A. (2013). Effect of gFe_2_O_3_ nanoparticles on photosynthetic characteristic of soybean (*Glycine max* (L.) Merr.): foliar spray versus soil amendment. *Acta Physiol. Plant. 35*, 3365–3375. doi: 10.1007/s11738-013-1369-8

Almutairi, Z.M. (2016). Effect of nano-silicon application on the expression of salt tolerance genes in germinating tomato (*Solanum lycopersicum*L.) seedlings under salt stress. *Plant Omics 9*, 106–114. doi: *10.3316/informit.888088806058398*

Apodaca, A., Tan, W., Dominguez, O.E., Hernandez-Viezcas, J.E., Peralta-Videa, J.R., Gardea-Torresdey, J.L. (2017). Physiological and biochemical effects of nanoparticulate copper, bulk copper, copper chloride, and kinetin in kidney bean (*Phaseolus vulgaris*) plants. *Sci. Total Environ. 599*, 2085–2094. doi: [10.1016/j.scitotenv.2017.05.095](https://doi.org/10.1016/j.scitotenv.2017.05.095)

Askary, M., Talebi, S.M., Amini, F., Bangan, A.D. (2017). Effects of iron nanoparticles on *Mentha piperita* L. under salinity stress. *Biologia*  *63*, 65–75. doi: [10.6001/biologija.v63i1.3476](https://doi.org/10.6001/biologija.v63i1.3476)

Avestan, S., Ghasemnezhad, M., Esfahani, M., Byrt, C.S. (2019). Application of nano-silicon dioxide improves salt stress tolerance in strawberry plants. *Agronomy 9*: 246. doi: [10.3390/agronomy9050246](https://doi.org/10.3390/agronomy9050246)

Behboudi, F., Tahmasebi-Sarvestani, Z., Kassaee, M.Z., Modares-Sanavi, S.A.M., Sorooshzadeh, A., Ahmadi, S.B. (2018). Evaluation of chitosan nanoparticles effects on yield and yield components of barley (*Hordeum vulgare* L.) under late season drought stress*. J. Water. Environ. Nanotechnol. 3*, 22–39. doi: [10.22090/JWENT.2018.01.003](https://dx.doi.org/10.22090/jwent.2018.01.003)

Canas, J.E., Long, M., Nations, S., Vadan, R., Dai, L., Luo, M., Ambikapathi, R., Lee, E.H., Olszyk, D. (2008). Effects of functionalized and non functionalized single-walled carbon nanotubes on root elongation of select crop species. *Environ. Toxicol. Chem.* *27*, 1922–1931. doi: [10.1897/08-117.1](https://doi.org/10.1897/08-117.1)

Cao, Z., Rossi, L., Stowers, C., Zhang, W., Lombardini, L., Ma, X. (2018). The impact of cerium oxide nanoparticles on the physiology of soybean (*Glycine max* (L.) Merr.) under different soil moisture conditions. *Environ. Sci. Pollut. Res. 25*, 930–939. doi: [10.1007/s11356-017-0501-5](https://doi.org/10.1007/s11356-017-0501-5)

Chandra, S., Pradhan, S., Mitra, S., Patra, P., Bhattacharya, A., Pramanik, P., Goswami A. (2014). High throughput electron transfer from carbon dots to chloroplast: a rationale of enhanced photosynthesis. *Nanoscale 6*, 3647–3655. doi: [10.1039/C3NR06079A](https://doi.org/10.1039/C3NR06079A)

Chen, Q., Chen, L., Nie, X., Man, H., Guo, Z., Wang, X., Tu, J., Jin, G., Ci, L. (2020) Impacts of surface chemistry of functional carbon nanodots on the plant growth. *Ecotoxicol. Environ. Safety* *206*, 111220. doi: 10.1016/j.ecoenv.2020.111220

Delfani, M., Baradarn-Firouzabadi, M., Farrokhi, N., Makarian, H. (2014). Some physiological responses of black-eyed pea to iron and magnesium nanofertilizers. *Commun. Soil Sci. Plant Anal. 45*, 530–540. doi: [10.1080/00103624.2013.863911](https://doi.org/10.1080/00103624.2013.863911)

Djanaguiraman, M., Belliraj, N., Bossmann, S.H., Prasad, P.V.V. (2018a). High-temperature stress alleviation by selenium nanoparticle treatment in grain sorghum. *ACS Omega* *3*, 2479–2491.doi: [10.1021/acsomega.7b01934](https://doi.org/10.1021/acsomega.7b01934)

El-Batal, A.I., Gharib, F.A.E.L., Ghazi, S.M., Hegazi, A.Z., Hafz, A.G.M.A.E. (2016). Physiological responses of two varieties of common bean (*Phaseolus vulgaris* L.) to foliar application of silver nanoparticles. *Nanomater. Nanotechnol*. *6*, 13. doi: [10.5772/62202](https://doi.org/10.5772%2F62202)

Elsheery, N.I., Sunoj, V.S.J., Wen, Y., Zhu, J.J., Muralidharan, G., Cao, K.F. (2020). Foliar application of nanoparticles mitigates the chilling effect on photosynthesis and photoprotection in sugarcane. *Plant Physiol. Biochem*. *149*, 50-60. doi: [10.1016/j.plaphy.2020.01.035](https://doi.org/10.1016/j.plaphy.2020.01.035)

Faizan, M., Faraz, A., Yusuf, M., Khan, S.T., Hayat, S. (2018). Zinc oxide nanoparticle-mediated changes in photosynthetic efficiency and antioxidant system of tomato plants, *Photosynthetica 56*, 678–686. doi: 10.1007/s11099-017-0717-0

Faraji, J., Sepehri, A. (2020). Exogenous nitric oxide improves the protective effects of TiO_2_ nanoparticles on growth, antioxidant system, and photosynthetic performance of wheat seedlings under drought stress. *J. Soil. Sci. Plant Nutr*. *20*, 703–714. doi: 10.1007/s42729-019-00158-0

Ghafariyan, M.H., Malakouti, M.J., Dadpour, M.R., Stroeve, P., Mahmoudi, M. (2013). Effects of magnetite nanoparticles on soybean chlorophyll. *Environ. Sci. Technol. 47*, 10645–10652. doi: 10.1021/es402249b

[Ghorbanpour](https://pubs.rsc.org/en/results?searchtext=Author%3AMansour%20Ghorbanpour), M., [Mohammadi](https://pubs.rsc.org/en/results?searchtext=Author%3AHamid%20Mohammadi), H.,  [Kariman](https://pubs.rsc.org/en/results?searchtext=Author%3AKhalil%20Kariman), K. (2020). Nanosilicon-based recovery of barley (*Hordeum vulgare*) plants subjected to drought stress. ***Environ. Sci.: Nano* *7*,** 443-461. doi: [10.1039/C9EN00973F](https://doi.org/10.1039/C9EN00973F)

Gui, X., Rui, M., Song, Y., Ma, Y., Rui, Y., Zhang, P., Liu, L. (2017). Phytotoxicity of CeO_2_ nanoparticles on radish plant (*Raphanus sativus*). *Environ. Sci. Pollut. Res*. *24*, 13775–13781. doi: [10.1007/s11356-017-8880-1](https://doi.org/10.1007/s11356-017-8880-1)

Gui, X., Zhang, Z., Liu, S., Ma, Y., Zhang, P., He, X., Li, Y., Zhang, J., Li, H., Rui, Y., Liu, L., Cao, W. (2015). Fate and phytotoxicity of CeO_2_ nanoparticles on lettuce cultured in the potting soil environment. *PLoS One 10*, e0134261. doi: [10.1371/journal.pone.0134261](https://dx.doi.org/10.1371%2Fjournal.pone.0134261)

Haghighi, M., Abolghasemi, R., da Silva, J.A.T. (2014). Low and high temperature stress affect the growth characteristics of tomato in hydroponic culture with Se and nano-Se amendment. *Sci. Hortic*. *178*, 231–240. doi: [10.1016/j.scienta.2014.09.006](https://doi.org/10.1016/j.scienta.2014.09.006)

Hernandez, H.H., Benavides-Mendoza, A., Ortega-Ortiz, H., Hernandez-Fuentes, A.D., Juárez-Maldonado, A. (2017). Cu Nanoparticles in chitosan-PVA hydrogels as promoters of growth, productivity and fruit quality in tomato. *Emir. J. Food Agric*. *29*, 573–580. doi: 10.9755/ejfa.2016-08-1127

Hojjat, S.S., Kamyab, M. (2017). The effect of silver nanoparticle on Fenugreek seed germination under salinity levels. *Russ. Agric. Sci. 43*, 61–65. doi: 10.3103/S1068367417010189

Iqbal, M.A. (2019). *Nano-fertilizers for Sustainable Crop Production under Changing Climate: A Global Perspective*. London, U.K.: Sustainable Crop Production London, U.K. IntechOpen. p. 293–303. doi: 10.5772/intechopen.89089

Jaberzadeh, A., Moaveni, P., Moghadam, H.R.T., Zahedi, H. (2013). Influence of bulk and nanoparticles titanium foliar application on some agronomic traits, seed gluten and starch contents of wheat subjected to water deficit stress. *Notulae. Botanicae. Horti. Agrobotanici*. *41*, 201–207. doi: [10.15835/nbha4119093](https://doi.org/10.15835/nbha4119093)

Jeyasubramanian, K., Thoppey, U.U.G., Hikku, G.S., Selvakumar, N., Subramania, A., Krishnamoorthy, K. (2016). Enhancement in growth rate and productivity of spinach grown in hydroponics with iron oxide nanoparticles. *RSC Adv. 6*, 15451–15459. doi: [10.1039/C5RA23425E](https://doi.org/10.1039/C5RA23425E)

Khan, M.N. (2016). Nano-titanium Dioxide (Nano-TiO_2_) mitigates NaCl stress by enhancing antioxidative enzymes and accumulation of compatible solutes in tomato (*Lycopersicon esculentum* Mill.). *J. Plant Sci*. *11*, 1–11. doi: [10.3923/jps.2016.1.11](http://dx.doi.org/10.3923/jps.2016.1.11)

Kole, C., Kole, P., Randunu, K.M., Choudhary, P., Podila, R., Ke, P.C., Rao, A.M., Marcus, R.K. (2013). Nanobiotechnology can boost crop production and quality: first evidence from increased plant biomass, fruit yield and phytomedicine content in bitter melon (*Momordica charantia*). *BMC Biotechnol.* *13*, 37. doi: 10.1186/1472-6750-13-37

Konate, A., Yao, W.Y., Xiao, H., Adeel, M., Peng, Z., Hui, M.Y., Yun, D.Y., Zhe, Z.J., Jie, Y., Kizito, S., Kui, R.Y., Yong, Z.Z. (2018). Comparative effects of nano and bulk-Fe_3_O_4_ on the growth of cucumber (*Cucumis sativus*). *Ecotoxicol. Environ. Safety 165*, 547–554. doi: [10.1016/j.ecoenv.2018.09.053](https://doi.org/10.1016/j.ecoenv.2018.09.053)

Kumar, V., Guleria, P., Kumar, V., Yadav, S.K. (2013). Gold nanoparticle exposure induces growth and yield enhancement in *Arabidopsis thaliana*. *Sci. Total Environ*. *461*, 462–468. doi: [10.1016/j.scitotenv.2013.05.018](https://doi.org/10.1016/j.scitotenv.2013.05.018)

Larue, C., Castillo-Michel, H., Sobanska, S., Cecillon, L., Bureau, S., Barthes, V., Querdane, L., Carriere, M., Sarret, G. (2014). Foliar exposure of the crop *Lactuca sativa* to silver nanoparticles: evidence for internalization and changes in Ag speciation. *J. Hazard. Mater*. *264*, 98–106. doi: [10.1016/j.jhazmat.2013.10.053](https://doi.org/10.1016/j.jhazmat.2013.10.053)

Le, V.N., Rui, Y., Gui, X., Li, X., Liu, S., Han, Y. (2014). Uptake, transport, distribution and bio-effects of SiO_2_ nanoparticles in Bt-transgenic cotton. *J. Nanobiotech*. 12, 50. doi: 10.1186/s12951-014-0050-8

Li, J., Hu, J., Ma, C., Wang, Y., Wu, C., Huang, J., Xing, B. (2016). Uptake, translocation and physiological effects of magnetic iron oxide (g-Fe_2_O_3_) nanoparticles in corn (*Zea mays* L.). *Chemosphere 159*, 326–334. doi: [10.1016/j.chemosphere.2016.05.083](https://doi.org/10.1016/j.chemosphere.2016.05.083)

Lin, S., Reppert, J., Hu, Q., Hudson, J.S., Reid, M.L., Ratnikova, T.A., Rao, A.M., Luo, H., Ke, P.C. (2009). Uptake, translocation, and transmission of carbon nanomaterials in rice plants. *Small* *5*, 1128–1132. doi: [10.1002/smll.200801556](https://doi.org/10.1002/smll.200801556)

Lopez-Moreno, M.L., de la Rosa, G., Cruz-Jiménez, G., Castellano, L., Peralta-Videa, J.R., Gardea-Torresdey, J.L. (2017). Effect of ZnO nanoparticles on corn seedlings at different temperatures; X-ray absorption spectroscopy and ICP/OES studies. *Microchem. J.* 134, 54–61. doi: 10.1016/j.microc.2017.05.007

Lu, L., Huang, M., Huang, Y., Corvini, P.F.X., Ji, R., Zhao, L. (2020). Mn_3_O_4_ nanozymes boost endogenous antioxidant metabolites in cucumber (Cucumis sativus) plant and enhance resistance to salinity stress. ***Environ. Sci.: Nano 7***, 1692-1703. doi: [10.1039/D0EN00214C](https://doi.org/10.1039/D0EN00214C)

Ma, C., Liu, H., Chen, G., Zhao, Q., Guo, H., Minocha, R., Long, S., Tang, Y., Saad, E.M., DeLaTorreRoche, R., White, J.C., Xing, B., Dhankher, O.P. (2020). Dual roles of glutathione in silver nanoparticle detoxification and enhancement of nitrogen assimilation in soybean (Glycine max (L.) Merrill). ***Environ. Sci.: Nano 7***, 1954-1966. doi: [10.1039/D0EN00147C](https://doi.org/10.1039/D0EN00147C)

Mahmoud, L.M., Dutt, M., Shalan, A.M., El-Kady, M.E., El-Boray, M.S., Shabana, Y.M., Grosser, J.W. (2020). Silicon nanoparticles mitigate oxidative stress of in vitro derived banana (*Musa acuminata* ‘Grand Nain’) under simulated water deficit or salinity stress. *South Afr. J. Bot. 132*, 155–163. doi: [10.1016/j.sajb.2020.04.027](https://doi.org/10.1016/j.sajb.2020.04.027)

Mohammadi, R., Maali-Amiri, R., Abbasi, A. (2013). Effect of TiO_2_ nanoparticles on chickpea response to cold stress. *Biol. Trace Elem. Res*. *152*, 403–410. doi: 10.1007/s12011-013-9631-x

Mozafari, A.A., Havas, F., Ghaderi, N. (2018). Application of iron nanoparticles and salicylic acid in *in vitro* culture of strawberries (*Fragaria* x *ananassa*Duch.) to cope with drought stress. *Plant Cell Tissue Organ Cult.* *132*, 511–523. doi: 10.1007/s11240-017-1347-8

Mukherjee, A., Sun, Y., Morelius, E., Tamez, C., Bandyopadhyay, S., Niu, G., White, J.C., Peralta-Videa, J.R., Gardea-Torresdey, J.L. (2016). Differential toxicity of bare and hybrid ZnO nanoparticles in green pea (*Pisum sativum* L.): A life cycle study. *Front. Plant Sci. 6*, 1242. doi: [10.3389/fpls.2015.01242](https://doi.org/10.3389/fpls.2015.01242)

Mustafa, G., Komatsu, S. (2016). Insights into the response of soybean mitochondrial proteins to various sizes of aluminum oxide nanoparticles under flooding stress. *J. Proteo. Res.*  *15*, 4464–4475. doi: [10.1021/acs.jproteome.6b00572](https://doi.org/10.1021/acs.jproteome.6b00572)

Poscic, F., Mattiello, A., Fellet, G., Miceli, F., Marchiol, L. (2016). Effects of cerium and titanium oxide nanoparticles in soil on the nutrient composition of barley (*Hordeum vulgare* L.) kernels. *Int. J. Environ. Res. Public Health 13*, 577. doi: [10.3390/ijerph13060577](https://dx.doi.org/10.3390%2Fijerph13060577)

Rajput, V., Minkina, T., Fedorenko, A., Sushkova, S., Mandzhieva, S., Lysenko, V., Duplii, N., Fedorenko, G., Dvadnenko, K., Ghazaryan, K. (2018a). Toxicity of copper oxide nanoparticles on spring barley (*Hordeum sativum* distichum). *Sci. Total Environ*. *645*, 1103-1113. doi: 10.1016/j.scitotenv.2018.07.211

Rezvani, N., Sorooshzadeh, A., Farhadi, N. (2012). Effect of nano-silver on growth of saffron in flooding stress. *World Acad. Sci. Eng. Technol*. *6*, 517–522. doi: [10.22048/JSAT.2014.6188](https://dx.doi.org/10.22048/jsat.2014.6188)

Rico, C.M., Johnson, M.G., Marcus, M.A., Andersen, C.P. (2017). Intergenerational responses of wheat (*Triticum aestivum* L.) to cerium oxide nanoparticles exposure. *Environ. Sci.: Nano 4,* 700–711. doi: [10.1039/C7EN00057J](https://doi.org/10.1039/C7EN00057J)

Rico, C.M., Morales, M.I., Barrios, A.C., McCreary, R., Hong, J., Lee,W.-Y., Nunez, J., Peralta-Videa, J.R., Gardea-Torresdey, J.L. (2013). Effect of cerium oxide nanoparticles on the quality of rice (*Oryza sativa* L.) grains. *J. Agric. Food Chem*. *61*, 11278–11285. doi: [10.1021/jf404046v](https://doi.org/10.1021/jf404046v)

Rui, M., Ma, C., Hao, Y., Guo, J., Rui, Y., Tang, X., Zhu, S. (2016). Iron oxide nanoparticles as a potential iron fertilizer for peanut (*Arachis hypogaea*). *Front. Plant Sci*. *7,* 815. doi: [10.3389/fpls.2016.00815](https://doi.org/10.3389/fpls.2016.00815)

Saharan, V., Kumaraswamy, R.V., Choudhary, R.C., Kumari, S., Pal, A., Raliya, R., Biswas, P. (2016). Cu-chitosan nanoparticle mediated sustainable approach to enhance seedling growth in maize by mobilizing reserved food. *J. Agricul. Food. Chem. 64*, 6148–6155. doi: [10.1021/acs.jafc.6b02239](https://doi.org/10.1021/acs.jafc.6b02239)

Shallan, M.A., Hassan, H.M., Namich, A.A., Ibrahim, A.A. (2016). Biochemical and physiological effects of TiO_2_ and SiO_2_ nanoparticles on cotton plant under drought stress. *Res. J. Pharma. Bio. Chem. Sci. 7*, 1540–1551.

Shams, G., Ranjbar, M., Amiri, A. (2013). Effect of silver nanoparticles on concentration of silver heavy element and growth indexes in cucumber (*Cucumis sativus* L. negeen). *J. Nanoparticle Res. 15*, 1630. doi: 10.1007/s11051-013-1630-5

Shekhawat, G.S., Mahawar, L., Rajput, P., Rajput, V.D., Minkina, T., Singh, R.K. (2021). Role of engineered carbon nanoparticles (CNPs) in promoting growth and metabolism of *Vigna radiata* (L.) Wilczek: insights into the biochemical and physiological responses. *Plants* *10*, 1317. doi: 10.3390/plants10071317

Sheykhbaglou, R., Sedghi, M., Shishevan, M.T., Sharifi, R.S. (2010). Effects of nano-iron oxide particles on agronomic traits of soybean. *Not. Sci. Biol*. *2*, 112–113. doi: 10.15835/nsb224667

Singh, A., Singh, N.B., Hussain, I., Singh, H. (2017). Effect of biologically synthesized copper oxide nanoparticles on metabolism and antioxidant activity to the crop plants *Solanum lycopersicum*and *Brassica oleracea* var. botrytis. *J. Biotechnol*. *262*, 11–27. doi: [10.1016/j.jbiotec.2017.09.016](https://doi.org/10.1016/j.jbiotec.2017.09.016)

Srivastava, A., Rao, D.P. (2014). Enhancement of seed germination and plant growth of wheat, maize, peanut, and garlic using multiwalled carbon nanotubes. *Eur. Chem. Bull*. *3*, 502-504.

Tarafdar, J.C., Raliya, R., Mahawar, H., Rathore, I. (2014). Development of zinc nanofertilizer to enhance crop production in pearl millet (*Pennisetum americanum*). *Agric. Res*. *3*, 257–262. doi: 10.1007/s40003-014-0113-y

Tombuloglu, H., Slimani, Y., Tombuloglu, G., Almessiere, M., Baykal, A. (2019). Uptake and translocation of magnetite (Fe_3_O_4_) nanoparticles and its impact on photosynthetic genes in barley (*Hordeum vulgare* L.). *Chemosphere 226*, 110-122. doi: [10.1016/j.chemosphere.2019.03.075](https://doi.org/10.1016/j.chemosphere.2019.03.075)

Torre-Roche, R.D.L., Hawthorne, J., Deng, Y., Xing, B., Cai, W., Newman, L.A., Wang, Q., Ma, X., Hamdi, H., White, J.C. (2013). Multiwalled carbon nanotubes and C_60_ fullerenes differentially impact the accumulation of weathered pesticides in four agricultural plants. *Environ. Sci. Technol.* *47*, 12539–12547. doi: [10.1021/es4034809](https://doi.org/10.1021/es4034809)

Tripathi, D.K., Singh, S., Singh, V.P., Prasad, S.M., Chauhan, D.K., Dubey, N.K. (2016). Silicon nanoparticles more efficiently alleviate arsenate toxicity than silicon in maize cultivar and hybrid differing in arsenate tolerance. *Front. Environ. Sci*. *4,* 46. doi: [10.3389/fenvs.2016.00046](https://doi.org/10.3389/fenvs.2016.00046)

Tripathi, D.K., Singh, S., Singh, V.P., Prasad, S.M., Dubey, N.K., Chauhan, D.K. (2017). Silicon nanoparticles more effectively alleviated UV-B stress than silicon in wheat (*Triticum aestivum*) seedlings. *Plant Physiol. Biochem*. *110*, 70–81. doi: [10.1016/j.plaphy.2016.06.026](https://doi.org/10.1016/j.plaphy.2016.06.026)

Venkatachalam, P., Priyanka, N., Manikandan, K., Ganeshbabu, I., Indiraarulselvi, P., Geetha, N., Sahi, S.V. (2017). Enhanced plant growth promoting role of phycomolecules coated zinc oxide nanoparticles with P supplementation in cotton (*Gossypium hirsutum* L.). *Plant Physiol. Biochem. 110*, 118–127. doi: [10.1016/j.plaphy.2016.09.004](https://doi.org/10.1016/j.plaphy.2016.09.004)

Wang, H., Kou, X., Pei, Z., Xiao, J.Q., Shan, X., Xing, B. (2011). Physiological effects of magnetite (Fe_3_O_4_) nanoparticles on perennial ryegrass (*Lolium perenne* L.) and pumpkin (*Cucurbita mixta*) plants. *Nanotoxicol*. *5,* 30–42. doi: 10.3109/17435390.2010.489206

Xu, J., Luo, X., Wang, Y., Feng, Y. (2018). Evaluation of zinc oxide nanoparticles on lettuce (*Lactuca sativa* L.) growth and soil bacterial community. *Environ. Sci. Pollut. Res*. *25*, 6026–6035. doi: [10.1007/s11356-017-0953-7](https://doi.org/10.1007/s11356-017-0953-7)

Younis, A.A., Khattab, H., Emam, M.M. (2020). Impacts of silicon and silicon nanoparticles on leaf ultrastructure and *TaPIP1* and *TaNIP2* gene expressions in heat stressed wheat seedlings. *Biol. Plant*. *64*, 343-352. doi: 10.32615/bp.2020.030

Yusefi-Tanha, E., Fallah, S., Rostamnejadi, A., Pokhrel, L.R. (2020). Particle size and concentration dependent toxicity of copper oxide nanoparticles (CuONPs) on seed yield and antioxidant defense system in soil grown soybean (*Glycine max* cv. Kowsar). *Sci. Total. Environ*. *715*, 136994. doi: [10.1016/j.scitotenv.2020.136994](https://doi.org/10.1016/j.scitotenv.2020.136994)
